# Clinical outcomes of combined cataract surgery with iStent *inject*^®^ W implantation versus cataract surgery alone in patients with open-angle glaucoma: real life data in a community-based outpatient setting

**DOI:** 10.1007/s00417-025-06909-3

**Published:** 2025-08-27

**Authors:** Fidan A. Aghayeva, Abdelrahman Assaf, Shakriiar Gurbanzade, Ines M. Lanzl

**Affiliations:** 1 Technical University of Munich, Munich, Germany; 2 Chiemsee Eye Clinic, Prien on Chiemsee, Germany; 3 Department of Ophthalmology, Technical University of Munich, Ismaninger Str. 22, Munich, 81675 Germany

**Keywords:** Cataract surgery, Glaucoma, Intraocular pressure, IOP-lowering medications, iStent, Minimally invasive glaucoma surgery

## Abstract

**Purpose:**

This study aims to evaluate and compare intraocular pressure (IOP) change and topical IOP-lowering medication use after combined cataract surgery with iStent *inject*^®^ W implantation versus cataract surgery alone in patients with open-angle glaucoma.

**Methods:**

Retrospective comparative chart review of 150 patients (150 eyes) with different types of open-angle glaucoma, who were naïve to any previous ocular procedure and underwent combined cataract surgery with iStent^®^ W implantation (100 eyes) or cataract surgery alone (50 eyes). Study outcomes were median IOP change and the mean number of topical IOP-lowering medications at 3, 6 and 12 months after surgery.

**Results:**

The median IOP change in the iStent inject^®^ W group at 3-, 6- and 12 months follow-up was − 4 (-6 to -2) mmHg (*p* < 0.001), -4 (-6 to -1) mmHg (*p* < 0.001), and − 4 (-6.75 to -2) mmHg (*p* < 0.001), respectively. The median IOP change in the control group at 3-, 6- and 12 months follow-up was 0 (-1.25 to 2) mmHg, 1 (-1 to 3) mmHg, and 0 (-2.5 to 3) mmHg, respectively. IOP reduction in the study group was statistically significantly larger than in the control group at all follow-ups (*p* < 0.001). We found a statistically significant difference between the mean number of topical IOP-lowering medications before and at 12 months in the combined surgery group (*p* < 0.001) as compared to the control group.

**Conclusions:**

This study shows a statistically significant IOP reduction at 3-, 6- and 12 months and reduction of topical IOP-lowering medications at 12 months after combined cataract surgery with iStent *inject*^®^ W implantation.

**Supplementary Information:**

The online version contains supplementary material available at 10.1007/s00417-025-06909-3.

## Key messages

*****What is known***:**

- Microinvasive glaucoma surgeries (MIGS) are a less invasive yet clinically effective alternative for the surgical management of open angle glaucoma.
- iStent *inject*^®^ W implantation combined with cataract surgery leads to statistically significant intraocular pressure (IOP) reduction.

*****What is new***:**

- Cataract surgery combined with iStent inject^®^ W implantation diminishes significantly the IOP and the number of topical IOP medications for up to 12 months.
- The IOP lowering effect of cataract surgery combined withiStent inject^®^ W is correlated positively with the preoperative IOP: the higher the preoperative IOP, the higher the IOP decrease.

**Supplementary Information:**

The online version contains supplementary material available at 10.1007/s00417-025-06909-3.

## Introduction

Microinvasive glaucoma surgeries (MIGSs) are modern alternative procedures in the surgical treatment of open angle glaucoma [1–3]. The first trabecular micro-bypass glaucoma stent from non-ferromagnetic heparin-coated titanium, iStent^®^ (Glaukos Corporation, Laguna Hills, CA, USA), was introduced in 1999, with the first implantation in Europe and in USA in 2003 and in 2005, respectively. The principal mechanism of the intraocular pressure (IOP) lowering is achieved by creating a direct bypass channel through trabecular meshwork (TM), between the anterior chamber and Schlemm’s canal (SC). The first-generation iStent^®^ with 1 mm length, 0.33 mm height and 60 µg weight received FDA (the United States Food and Drug Administration) approval in 2012. The US iStent Study Group results of the first prospective, multicenter randomized controlled trial were presented by Samuelson et al. in 2011. A total of 240 eyes across 29 investigational centers with mild to moderate open-angle glaucoma with IOP ≤ 24 mmHg controlled on one to three IOP-lowering drops were randomized to undergo combined cataract surgery with iStent^®^ implantation or cataract surgery alone. 72% of study eyes versus 50% of control eyes achieved unmedicated IOP ≤ 21 mmHg at one-year follow-up. An average medication reduction was 1.4 and 1.0 for the iStent^®^ and cataract-only group, respectively [4]. In 2012 Craven et al. published 2 years follow-up results from ongoing US iStent Study with the conclusion that IOP reduction on fewer medications was statistically significantly greater after combined cataract surgery with iStent implantation (a total of 53% of iStent^®^ eyes compared with 44% of control eyes had IOP reduction ≥ 20% without medication) [5].

The second-generation model, iStent *inject*^®^ with 360 μm length, 230 μm width and 80 μm inner diameter, allowed simultaneous implantation of 2 stents and received its FDA approval in 2018 [6]. The third generation iStent *inject*^®^ W with its CE Mark approval in 2020 has a larger base diameter of 360 μm, and therefore, due to its superior positioning, leads to greater IOP reduction in comparison to the iStent *inject*^®^ [7]. Our review based on a PubMed search for ‘iStent^®^’, revealed more than 300 peer-reviewed articles, of which only 15 studies were conducted using the iStent inject^®^ W [6–27]. Therefore, further research is warranted to expand the already existing data. According to newly published data by Morita et al. IOP reduction accounted to 11.9% and mean medication burden decreased up to 46.8% at 12 months after combined phacoemulsification with iStent *inject*^®^ W [8]. In another retrospective study including 85 eyes with 6 months follow up reduction of IOP and decrease in the number of IOP-lowering medications was 19.3% and 22.4%, respectively [9].

The aim of this retrospective study is to evaluate and compare IOP change and topical IOP-lowering medication use after combined cataract surgery with iStent *inject*^®^ W implantation versus cataract surgery alone in patients with open-angle glaucoma in a community-based outpatient setting.

## Materials and methods

We performed a retrospective comparative chart review of 150 patients (150 eyes) with different types of mild to moderate open-angle glaucoma (83.3% primary open angle (POAG) and 16.6% pseudoexfoliation glaucoma (PEXG)), who underwent combined cataract surgery with iStent *inject*^®^ W implantation (study group − 100 eyes) or cataract surgery alone (control group − 50 eyes) from January 2022 to January 2023 at Chiemsee Eye Clinic, Germany. The study adhered to the tenets of the Declaration of Helsinki, all patients consented to the use of their data for study purposes and informed consent was obtained from all the patients before surgery.

Indication for combined surgery was presence of early or moderate open angle glaucoma with borderline target or uncontrolled IOP under mono- or combined (one to three IOP-lowering medications) therapy. Those patients who underwent only cataract surgery and had borderline target or uncontrolled preoperative IOP under mono- or combined therapy received additional IOP lowering medications before surgery and were operated with controlled IOP that reached the target level of IOP in 60% of cases. 40% of cases still showed borderline target IOP. We used NICE thresholds for glaucoma disease definition, which is based on analysis of the optic disc using Optical coherence tomography (Optopol, Copernicus REVO OCT), automated visual field examination using the Medmont M700 Automated Perimeter (MAP, Australia), and IOP measurements (Goldmann applanation tonometry) [28]. Eye-specific target IOP was defined by NICE and European Glaucoma Society guidance [28, 29]. Patients with only one functional eye (monocular patients) or patients who underwent any previous ocular procedure such as selective laser trabeculoplasty or any other intraocular surgery, as well as patients with corneal endothelial cell density below 1500 cells/mm^2^ or with macular changes (see below) were excluded from the study. We included only one eye (right eye) per patient from those who underwent cataract surgery alone or combined cataract surgery with iStent *inject*^®^ W implantation for both eyes.

Main outcome measures were postoperative IOP levels and median IOP change in the operated eye. Secondary outcome was the mean number of topical IOP-lowering medications during follow-up. Preoperative IOP was taken at the time of surgery indication. Postoperatively, IOP was measured at 3-, 6- and 12 months follow-up after surgery.

In addition to the slit lamp clinical examination, specular endothelial microscopy (Tomey, USA) and optical coherence tomography for the macula (OCT Macula, Optopol, Copernicus REVO OCT) were performed before surgery to meet the exclusion criteria and after surgery to exclude presence of possible postoperative complications such as corneal endothelial dysfunction and retinal macular changes.

### Surgical procedure

Surgery was performed under topical anesthesia. After standard phacoemulsification the anterior chamber was filled with an ophthalmic cohesive viscoelastic device to widen the nasal angle. After rotating the head of the patient 30° away from the surgeon, and the surgical microscope oculars 30° towards the surgeon, the iStent inserter was introduced to the anterior chamber through the same temporal 2.2 mm clear corneal incision and approached the nasal angle under direct gonioscopic view (Swan Jacobs Goniolens, Volk, USA). The implantation was performed by slightly pushing and puncturing of the TM and sliding of the stent into SC; 2 stents (iStent *inject*^®^ W, Glaukos Corporation, Laguna Hills, CA, USA) were implanted at a distance of 2 to 3 o’clock hours from each other. At the end of the surgery viscoelastic was thoroughly rinsed out of the anterior chamber.

Postoperative medications included prednisolone acetate 1% eye drops 4 times daily in the first week with a tapering regimen during four weeks (weekly 4-3-2-1 taper schedule) and moisturizing eye drops. The decision to discontinue or reduce the IOP-lowering drops, used before surgery, was made one month after surgery based on the target IOP.

### Statistical analysis

All statistical analysis was conducted with IBM^®^ SPSS^®^ Statistics (Statistical Package for the Social Sciences). Absolute and relative frequencies were computed for dichotomous data, continuous data are presented as mean ± standard deviation and median respective inter-quartile range. Paired Wilcoxon test was used to compare pre- and post-operative IOP, and pre- and postoperative number of medications in both groups. Correlations between the IOP change from baseline to follow-up in the treated eye was analyzed using Spearman’s rank correlation. Descriptive data was compared using Chi-Square and Mann-Whitney U tests.

## Results

The mean age of the patients in both groups was 74 ± 7.5 (54–90) and 71 ± 8.5 (52–88) years; 98 (65.3%) women and 52 (34.6%) men. There were no statistically significant differences between type and severity of glaucoma between the study and control groups. The demographic and clinical data of all patients in both groups is presented in Table 1.

**Table 1:** Demographic and clinical data of all patients

Data	Study group	Control Group *
	Cataract surgery with iStent inject^®^ W	Cataract surgery alone
Number of patients/eyes		
Mean age ± SD (range), years	100/100	50/50
Sex, female/male	74 ± 7.5 (54–90)	71 ± 8.5 (52–88)
Type of glaucoma	56 (56%)/44 (44%)	42 (84%)/8 (16%)
POAG	84 (84%)	41 (82%)
PEX	16 (16%)	9 (18%)
Mean duration of glaucoma, years	8.1 ± 8.5	7.6 ± 6.6
Severity of glaucoma		
mild	38 (38%)	18 (36%)
moderate	62 (62%)	32 (64%)
Presence of topical therapy in operated eye: before surgery/after surgery	84 (84%)/76 (76%)	43 (86%)/45 (90%)
Mean number of topical IOP lowering medications before and at 12 months after surgery:	1.23 ± 0.8/1.08 ± 0.8	1.26 ± 0.5/1.34 ± 0.7
** None** - before surgery/after surgery	16 (16%)/24 (24%)	7 (14%)/5 (10%)
** 1** - before surgery/after surgery	51 (51%)/50 (50%)	23 (46%)/25 (50%)
** 2** - before surgery/after surgery	25 (25%)/22 (22%)	15 (30%)/16 (32%)
** 3** - before surgery/after surgery	8 (8%)/4 (4%)	5 (10%)/4 (8%)
IOP_max_ (mmHg)	30.6 ± 9.0	29.7 ± 8.9
CCT (µm)	528.2 ± 34.3	532.3 ± 25.2
CDVA	0.6 ± 0.3	0.5 ± 0.3
MD (dB)	5.8 ± 5.5	6.2 ± 6.8
Mean ECD ± SD (cells/mm^2^): before surgery/after surgery	2281 ± 452/1998 ± 464	2318 ± 475/2035 ± 482

SD = standard deviation; POAG = primary open angle glaucoma; PEX glaucoma = pseudoexfoliative glaucoma; IOP_max_ = maximum baseline intraocular pressure; CCT = central corneal thickness; CDVA = corrected distance visual acuity; MD = mean deviation (Medmont perimetry); Mean ECD = mean endothelial cell density

* no statistically significant differences in study parameters between groups

**Fig. 1:**
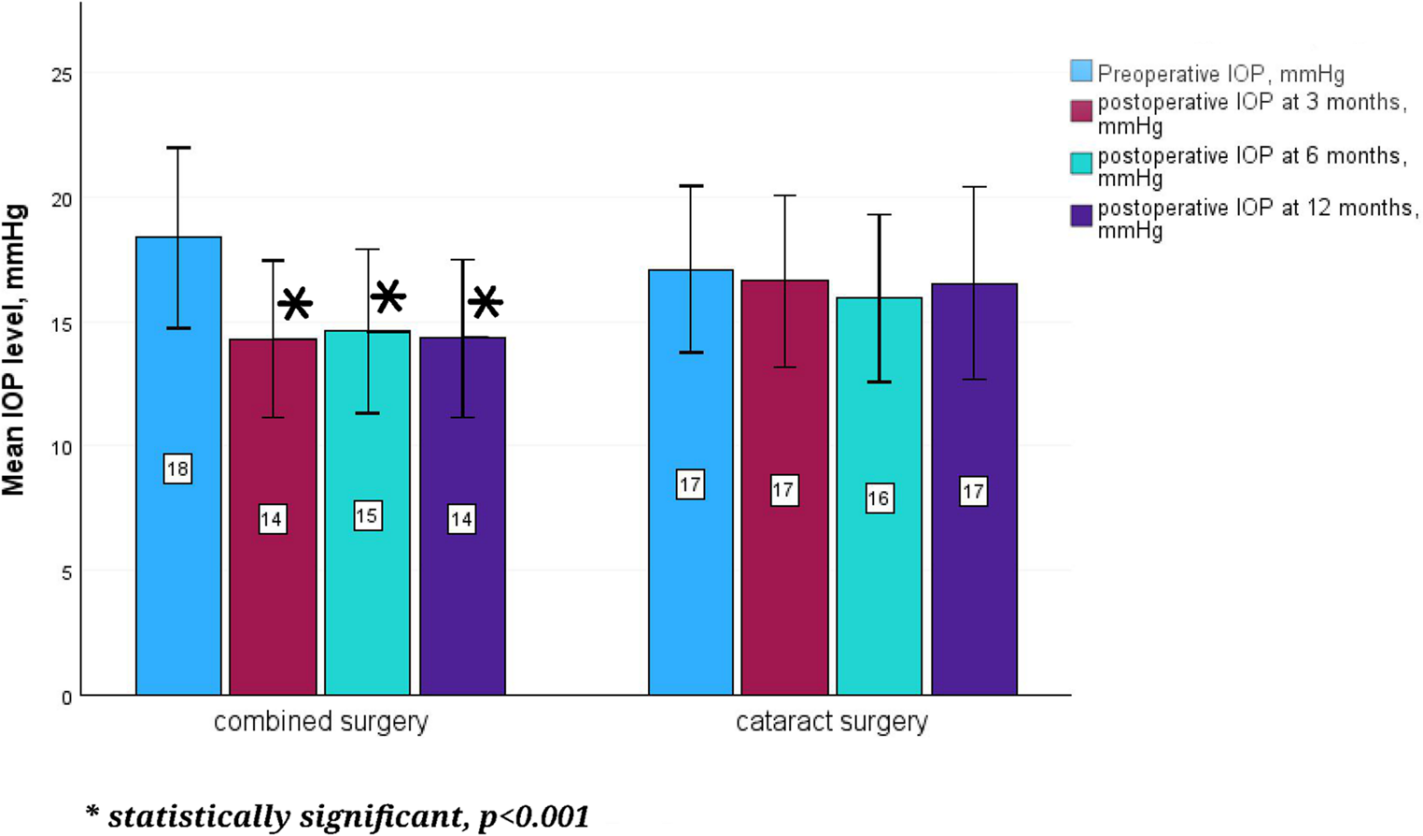
Mean IOP levels (mmHg) pre- and postoperatively in both groups. IOP = intraocular pressure. *statistically significant difference between pre- and postoperative IOP levels, *p* < 0.001

The median pre- and postoperative IOP in the study group at 3, 6 and 12 months were 18.5 (16–20) mmHg, 14.0.

(12-16.75) mmHg, 14.5 (12–16) mmHg and 14.5 (12–16) mmHg, respectively. The median pre- and postoperative IOP in the control group at 3, 6 and 12 months were 17 (15–20) mmHg, 17.0 (14–19) mmHg, 16 (13-18.25) mmHg and 16 (13.75-19) mmHg, respectively. The preoperative IOP was not significantly different between the study and control groups. The median postoperative IOP levels were statistically significantly lower than preoperative IOP levels at all follow-ups after combined surgery (−7.94, *p* < 0.001; −7.34, *p* < 0.001; −7.64, *p* < 0.001 at 3, 6 and 12 months, respectively), whereas in the control group no statistically significant pre- and postoperative IOP difference was revealed. The mean pre- and postoperative IOP levels in both groups are presented in Fig. 1. Distribution of eyes with different IOP levels pre- and postoperatively in both groups is presented in supplementary file.

The median IOP change after combined surgery at 3-, 6- and 12 months follow-up was − 4 (−6 to −2) mmHg, −4 (−6 to −1) mmHg, and − 4 (−6.75 to −2) mmHg, respectively. The median IOP change in the control group at 3-, 6- and 12 months follow-up was 0 (−1.25 to 2) mmHg, 1 (−1 to 3) mmHg, and 0 (−2.5 to 3) mmHg, respectively. The mean and percentage IOP changes in both groups at different follow-ups are presented in Table 2. Thus, IOP reduction in the study group was statistically significantly larger than in the control group at 3 months (−5.97, *p* < 0.001), 6 months (−4.63, *p* < 0.001), and 12 months (−5.24, *p* < 0.001) follow-up, respectively.

**Table 2:** Mean (mmHg) and percentage IOP (%) change at different follow-ups after surgery in both groups

Group	3 months		6 months		12 months	
	mean %		mean %		mean %	
Study Group	- 4.1 ± 3.6*	22.1*	- 3.7 ± 3.8*	20.4*	- 4 ± 3.7*	22*
Control Group	- 0.4 ± 2.9	2.3	- 1 ± 2.9	5.8	- 0.2 ± 3.9	1.2

IOP = intraocular pressure

*p<0.001; statistically significant difference between the both groups (Mann-Whitney Test)

**Fig. 2:**
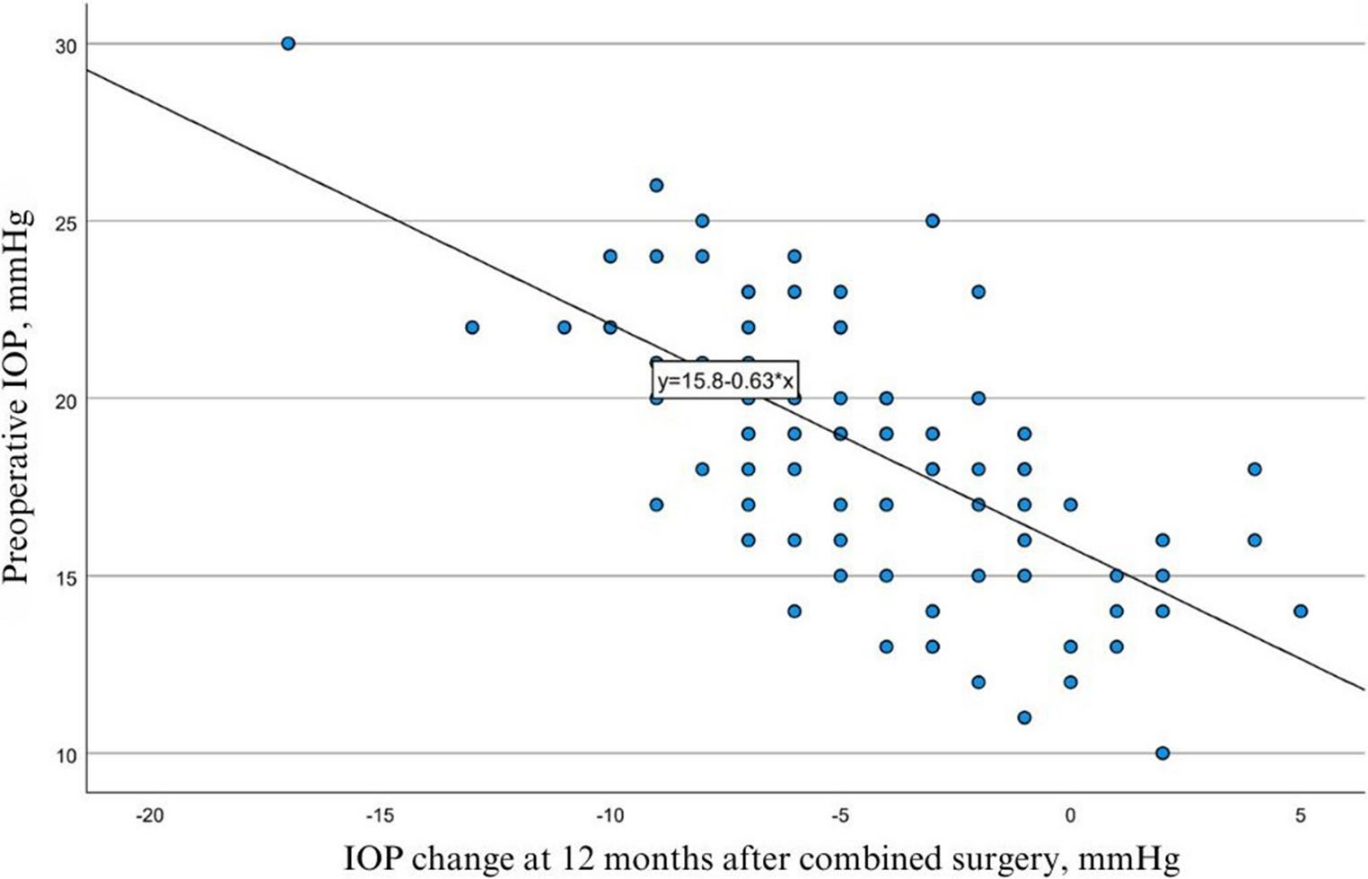
Correlation between preoperative IOP level in the operated eye and IOP change at 12 months follow-up after combined surgery (*p* < 0.001). IOP = intraocular pressure

The higher the preoperative IOP level was in the operated eye, the larger was the IOP-lowering effect at all follow-ups after combined surgery (−0.57, *p* < 0.001; −0.58, *p* < 0.001; −0.6, *p* < 0.001; at 3, 6 and 12 months, respectively) (Fig. 2).

We found a statistically significant difference between the mean number of topical IOP-lowering medications before and at 12 months after combined surgery (1.23 ± 0.8 vs. 1.08 ± 0.8; *p* < 0.001), but no significant difference was found between number of pre- and postoperative IOP-lowering medications in the control group (1.26 ± 0.5 vs. 1.34 ± 0.7). 24% of patients after combined cataract surgery with iStent *inject*^®^ W implantation vs. 16% before surgery did not need IOP-lowering medications. In the study group, 8% of the patients required three-fold antiglaucomatous therapy preoperatively, but only 4% required it 12 months after surgery. In the control group, the difference between the percentage of the patients who required three-fold IOP-lowering therapy preoperatively (10%) and 12 months after surgery (8%) was not significant (Table 1).

Transient IOP elevation in the first week after surgery was noted in 4% of cases in the study group and in 6% of cases in the control group. Stent malposition was detected in three eyes (3%) and hyphema with no requirement of additional interventions in 4 eyes (4%) in the study group, no hyphema was found in the control group. We did not have any cases of corneal endothelial dysfunction with severe corneal edema or corneal stromal scarring, persistent cystoid macular edema, choroidal effusions, Uveitis-Glaucoma-Hyphema Syndrome or endophthalmitis after surgery.

## Discussion

iStent^®^ *inject* W is the smallest implantable trabecular medical device approved for use in humans by the US FDA. Several studies have shown increased outflow facility after implantation of various iStent models. Bahler et al. found IOP reduction of 6.1 mmHg with one iStent and 9.7 mmHg with two, using anterior segment perfusion models [30]. Average IOP reduction following iStent inject^®^ W implantation in combination with cataract surgery is reported to be 1.04 mmHg – 2.7 mmHg (9-12.4%), and the reduction of IOP-lowering medications varies from 0.7 to 1.8 at 12 months follow-up after surgery [8, 9, 31–35]. The percentage IOP and medication reduction were 19.3% and 22.4%, respectively, at 6 months after combined cataract surgery with iStent inject^®^ W implantation [9]. According to newly published comparative iStent^®^/iStent^®^ *inject* W data of Morita et al. no significant difference in terms of the percent IOP reduction from baseline in iStent-treated eyes (8.0% reduction, *P* < 0.01) and iStent^®^ *inject* W-treated eyes (11.9% reduction, from the preoperative mean IOP of 15.0 ± 2.8 mmHg to the postoperative value of 13.8 ± 3.3 mmHg, *P* < 0.01) at 12 months after combined surgery was found. The mean medication reduction was 55% in iStent vs. 46.8% in iStent^®^ *inject* W group [8].

In our study mean IOP reduction was 3.7 ± 3.8 mmHg (20.4%) and 4 ± 3.7 mmHg (22%) at 6- and 12 months follow-up after combined surgery, respectively. Moreover, the larger IOP-lowering effect after combined surgery at different follow-ups in our study was associated with the higher preoperative IOP in the operated eye (*p* < 0.001). The mean reduction of topical IOP-lowering medications was 0.15 (12.2%) in the study group. Furthermore, target IOP was achieved in 24% of patients without IOP-lowering medications after combined surgery. Eye-specific target IOP was determined according to stage of disease/visual field damage at diagnosis, the baseline IOP and disease progression rate. IOP level of 18 to 20 mmHg with a reduction of at least 20% from baseline IOP, 15 to 17 mmHg with a reduction of at least 30% from baseline IOP, and 10 to 12 mmHg was defined as a target IOP in early, in moderate and in advanced glaucoma, respectively [29]. Onoe et al. reported a medication-free rate up to 69.4% in the iStent *inject* W-Phaco group at 12 months postoperatively [36]. The percentage of eyes requiring combination therapy with three IOP-lowering medications was 50% less at 12 months after combined surgery. However, there was no significant difference between the percentage of the patients who required three-fold IOP-lowering therapy preoperatively and 12 months after surgery in the control group. Among complications in iStent and control groups the following was reported by Craven et al.: elevated IOP in 3.4% and 4.3%; elevated IOP requiring oral or intravenous medications or surgery in 0.9% and 2.6%; conjunctival irritation from hypotensive medications in 0.9% and 2.6% cases; disc hemorrhage in 0.9% and 2.6%, respectively. However, their complication rate after combined surgery was not significantly higher compared to cataract surgery alone demonstrating a similar safety profile of combined surgery compared to cataract surgery [5]. In other iStent *inject* W-Phaco studies the incidence rates of transient IOP elevation and hyphema were reported at 2.4-6% and 1.8-6% [9, 35, 36].

In our study, transient IOP elevation in the first week after surgery was detected in 4% and in 6% of cases in the study and control groups, respectively, and hyphema occured in 4% of cases in the study group, which did not require additional intervention. Two iStent associated complications - stent obstruction and stent malposition were reported by Craven in 4.3% and 2.6% of all cases [5]. According to other published results, the rate of these stent related complications varied from 4 to 18% of cases [37–39]. In our study group, stent malposition was observed in 3% of cases. We found no sight-threatening complications of iStent implantation, such as corneal endothelial changes (endothelial failure with severe corneal edema and stromal scarring), retinal changes (persistent cystoid macular edema, development of subretinal fluid), choroidal effusions, Uveitis-Glaucoma-Hyphema Syndrome or endophthalmitis [7, 40–43].

The main strength of our study is that patients who underwent previous glaucoma procedures such as selective laser trabeculoplasty or other intraocular surgery were excluded from the study. Another strength of our community-based outpatient setting study is that we included one eye per patient to avoid bias in the results and possible MIGS dependent systemic effects like a consensual ophthalmotonic reaction [44]. The main limitations of our study are its retrospective design and the short follow-up. Thus, this study shows a statistically significant IOP reduction at 3-, 6- and 12 months follow-up after iStent *inject*^®^ W implantation in combination with cataract surgery in comparison to insignificant IOP change after cataract surgery alone in glaucoma patients. This MIGS approach is a clinically safe alternative surgical procedure that is associated with the reduction of topical IOP-lowering medication use at 12 months follow-up and therefore results in improved quality of life of patients with open angle glaucoma.

## Supplementary Information

Below is the link to the electronic supplementary material.

Supplementary file1 Distribution of eyes with different IOP levelspre- and postoperatively in both groups. IOP = intraocular pressure (PNG 85.7 KB)

## Data Availability

The authors confirm that the data supporting the findings of this study are available within the article and its supplementary materials. The results described in this manuscript are part of the MD thesis of Shakriiar Gurbanzade at the Technical University of Munich. The partial data that support the findings of this study was presented as a poster during EVER 2023 Congress and are openly available in Acta Ophthalmologica, Vol 102, № S279.
