# Supplementary figures and images for: Clinical outcomes of combined cataract surgery with iStent inject® W implantation versus cataract surgery alone in patients with open-angle glaucoma: real life data in a community-based outpatient setting
Source: Graefes Arch Clin Exp Ophthalmol. 2025 Aug 27;263(11):3191–8. doi: 10.1007/s00417-025-06909-3 (PMC12675655; doi:10.1007/s00417-025-06909-3)

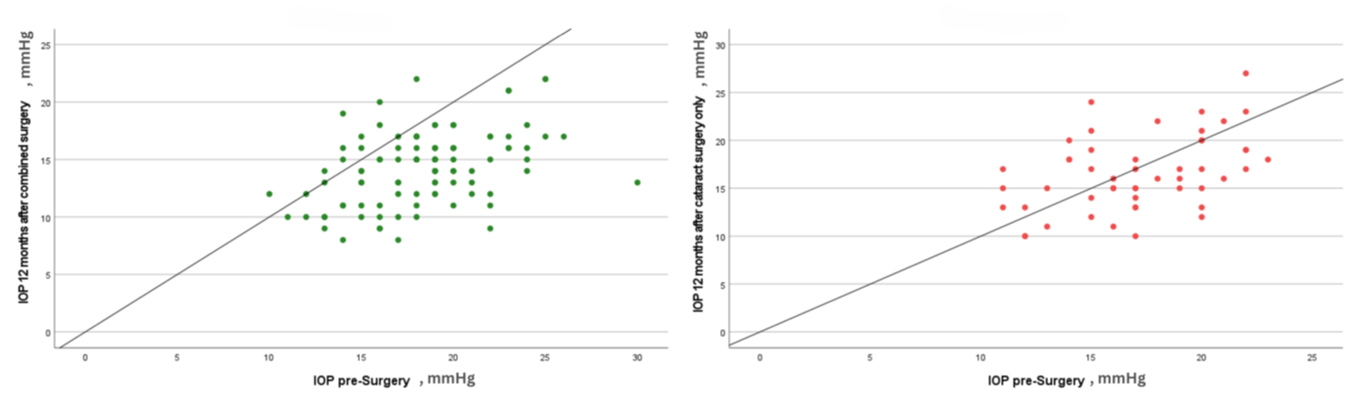

Supplement: Supplementary file 1 — Supplementary file1 Distribution of eyes with different IOP levelspre- and postoperatively in both groups. IOP = intraocular pressure (PNG 85.7 KB) [file 417_2025_6909_MOESM1_ESM.png]
